# Harnessing the power of probiotic strains in functional foods: nutritive, therapeutic, and next-generation challenges

**DOI:** 10.1007/s10068-024-01630-z

**Published:** 2024-06-20

**Authors:** Muneera Anwer, Ming Q. Wei

**Affiliations:** 1 Menzies Health Institute Queensland and School of Medical Science, Griffith University, Gold Coast Campus, Parklands Drive, Southport, QLD 4215 Australia; 2 Atta-ur-Rahman School of Applied Biosciences (ASAB), National University of Sciences and Technology (NUST), Islamabad, Pakistan

**Keywords:** Probiotic, Functional food, Health benefit, Food supplement, Microencapsulation

## Abstract

Functional foods have become an essential element of the diet in developed nations, due to their health benefits and nutritive values. Such food products are only called functional if they, “In addition to basic nutrition, have valuable effects on one or multiple functions of the human body, thereby enhancing general and physical conditions and/or reducing the risk of disease progression”. Functional foods are currently one of the most extensively researched areas in the food and nutrition sciences. They are fortified and improved food products. Presently, probiotics are regarded as the most significant and commonly used functional food product. Diverse probiotic food products and supplements are used according to the evidence that supports their strength, functionality, and recommended dosage. This review provides an overview of the current functional food market, with a particular focus on probiotic microorganisms as pivotal functional ingredients. It offers insights into current research endeavors and outlines potential future directions in the field.

## Introduction

In the last few years, the field of food production has been significantly transformed due to consumer demand. Consumers’ belief that food has a direct impact on well-being, and health has significantly contributed to a change in the field of food production. Nowadays, in addition to hunger satisfaction, food provides essential nutrients, prevents illnesses, and aids in the progression of consumers’ mental and physical health (Menrad, 2003). Functional foods play a remarkable contribution in this context. The need for this kind of food has increased considerably due to the high cost of medical centers, improving quality of life, and increasing life expectancy (Kotilainen et al., 2006). During the 1980s, the concept of functional foods originated in Japan (Valdemiro Carlos, 2011) which provides basic nutrition essential for living (Alissa and Ferns, 2012), helps in the welfare of physical and mental health, provides treatment and prevention from certain diseases, and enhances physiological functions (Lobo et al., 2010) like improving systemic circulation, immuno-potentiation, and aging control (Al-Sheraji et al., 2013). Functional food is defined as, “Food products that show resemblance to traditional food, but modified to have added health benefits to human and are consumed as a part of normal diet”.

Back in the earlier times, the advancement of functional food relied on food products strengthened with added minerals or/and vitamins, such as vitamin C, vitamin E, folic acid, calcium, iron, and zinc (Sloan, 2000). Later, the emphasis shifted to food products strengthened with added micronutrients like, phytosterol, soluble fiber, and omega-3 fatty acids to encourage better well-being or for the prevention of certain diseases like cancers (Sloan, 2002). Currently, further steps have been taken by food companies for the progression of new functional foods that provide multiple benefits to human health in one food product (Sloan, 2004). The physiological benefits of functional foods include, modification of the immune system, improving heart health, improved gastrointestinal system, healthy urinary tract system, reduce blood pressure, anti-inflammatory properties, antiviral and antibacterial activity, protection of vision, decreased osteoporosis, and reducing obesity. The functional property of these foods is based on biologically active components that are naturally present in the product and require optimizing health benefits by adding a particular ingredient. Recently, probiotics have acquired public attention due to their potential to improve human health. Due to this demand, the probiotic market has expanded rapidly worldwide. And as a result, they are used as supplements and as functional food raw materials (Chen et al., 2023), Table 1 represents major probiotic strains used in the market (Anwer et al., 2019; Ballini et al., 2023). This review will focus on the development of functional foods supplemented with probiotics, demonstrate the properties of probiotics, and cover all the possible treatment strategies currently in practice using probiotics by linking them with ongoing clinical studies. The future directions of probiotic functional food will also be discussed.

**Table 1:** Major probiotic strains used in the industry

Probiotics		
Lactic acid bacteria	Bifidobacterium	Other species
*Lactobacillus acidophilus*	*Bifidobacterium adolescentis*	*Aspergillus niger*
*Lactobacillus brevis*	*Bifidobacterium animalis*	*Aspergillus oryzue*
*Lactobacillus crispatus*	*Bifidobacterim bifidum*	*Bacillus cereus var. toyoi*
*Lactobacillus casei*	*Bifidobacterium breve*	*Clostridium butyricum*
*Lactobacillus delbrueckii* subsp. bulgaricus	*Bifidobacterium eriksonii*	*Escherichia coli* strain Nissle 1917 (EcN)
*Lactobacillus fermentum*	*Bifidobacterium infantis*	*Propionibacterium freudenreichii*
*Lactobacillus gasseri*	*Bifidobacterium longum*	*Saccharomyces cerevisiae*
*Lactobacillus gallinarum*	*Bifidobacterium lactis*	*Saccharomyces boulardii*
*Lactobacillus helveticus*	*Bifidobacterium pseudolongum*	*Saccharomyces bayanus*
*Lactobacillus johnsonii*	*Bifidobacterium thermophilum*	*Streptococcus thermophilus*
*Lactococcus lactis*		
*Lactobacillus plantarum*		
*Lactobacillus paracasei*		
*Lactobacillus reuteri*		
*Lactobacillus rhamnosus*		
*Lactobacillus salivarius*		
*Leuconostoc citreum*		
*Leuconostoc mesenteroides*		
*Pediococcus acidilactici*		
*Streptococcus thermophillus*		
*Enterococcus durans*		
*Enterococcus faecalis*		
*Enterococcus faecium*		

### Probiotics as a functional food

The notion of probiotics was established by scientist Elie Metchnikoff during the 1900s. He discovered that consuming live microbes such as *Lactobacillus bulgaricus* available in yogurt or fermented milk show improvement in several gastrointestinal (GIT) properties (De Simone, 2019). Recently, probiotics are defined as “living microbes which confer a health benefit to the host, upon ingestion in a tolerable amount” by the World Health Organization (WHO) and the Food and Agricultural Organization (FAO) of the United Nations (Hill et al., 2014). According to International Association for Scientific Prebiotics and Probiotics (ISAPP), certain minimum requirements must be fulfilled by probiotic microorganisms to be included in the category of functional food. Criteria such as evidence about their genus, species, and strain identifications; a valid scientific nomenclature for designation of strain; being deposited in an international culture collection; and having health benefits that must be validated by human study (at least one). Furthermore, a probiotics-containing functional food product must contain enough live microorganisms to offer the demanded positive health effects until the expiry date of the food product (Fusco et al., 2022). Moreover, probiotics should be adaptable to adverse GIT conditions, exhibit antagonistic effects against pathogenic microorganisms, have proven health benefits on the host, stimulate the immune system, and maintain their potency when exposed to certain food processing conditions (Plaza-Diaz et al., 2019). *Bifidobacterium* and Lactic acid bacteria are the most commonly studied and extensively used bacteria in the area of probiotics, however some different genera, for instance, *Streptococcus*, *Enterococcus*, *Saccharomyces*, and *Bacillus* have also been commercialized (Sarao and Arora, 2017). One of the most studied genera is *Lactobacillus*, and the most recent update reported 23 novel genera of *Lactobacillus.* This has sparked a new wave of scientific inquiry and their role as probiotics needs to be further explored and verified (Zheng et al., 2020). They are the normal flora of the colon. Microflora present in the colon performs several unique functions, and evaluating these functions is essential (Roberfroid et al., 1995). For the testing and development of functional foods, the gut is an obvious and natural target since it operates as a midway connection between metabolic pathways and the diet of human. For application in the dairy industry, an ideal probiotic must be acid and bile-resistant, perform modulation of the immune system, attach to epithelial cells of humans, produce antimicrobial compounds, grow exponentially, and produce health benefits to humans (Gorbach and Newton, 1996). Though the mechanisms of action of probiotics are specific to certain strains and it is diverse (Hill et al., 2014; Plaza-Diaz et al., 2019). Certain prebiotic substances serve as a stimulant for the growth of probiotics in the gut. Prebiotics like lactitol, inulin, lactulose, and xylitol are currently on the market due to their claimed benefits to boost the effect of probiotics (Zubillaga et al., 2001).

### Global statistics and therapeutic dosage

Currently, functional food is made up of vitamins, minerals, probiotics, and prebiotics which are now in human use in the form of certain products like yogurts, fermented milk baby foods, sports drinks, chewing gum, and confectionery. More recently, gut health functional food products particularly probiotics has dominated the food market in Europe and Japan with the launch of 379 products globally in 2005, several examples of commercial probiotic products with their origin are listed in Table 2 (Kaur and Das, 2011; Siró et al., 2008; Vergari et al., 2010). The growing interest of people in lifestyle and health, as well as issues associated with digestive and metabolic disorders, are essential factors in the contribution to expanding the probiotics industry (Elshaghabee et al., 2017), which accounts for a significant portion of the functional food market (Sarao and Arora, 2017). The global probiotics market in 2019 had a value of $48.4 billion and is predicted to rise at a compound annual growth rate (CAGR) of 7.4% from 2019 to 2024 (BBC, 2020). Asia now has the largest market for probiotic products, as well as the highest growth rate (Intelligence, 2019), indicating that there are intriguing demands to be investigated (Kwak, 2014). The practice of using probiotics in food products or as a supplement having a particular health benefit requires prior human experiments, such as randomized clinical trials, positive meta-analyzes, or a strong suggestion from observational research studies (Hill et al., 2014). The recommended amount of probiotic microorganisms varies depending on the strain and product, stated by World Gastroenterology Organization (WGO) (Organisation, 2017). Even though numerous commercially accessible products contain a probiotic dose in the range of 1 to 10 billion colony-forming units (CFU), few formulations have been demonstrated to be effective at a low level of dose, whereas others need higher doses (Sarao and Arora, 2017). Upon regular intake of 100 mL or 100 g of probiotic food, it is advised that probiotic formulae should have at least 10^6^–10^7^ CFU per gram of probiotic food, otherwise a total of 10^8^–10^9^ CFU, to have the therapeutic potential (Flach et al., 2018).

**Table 2:** Examples of some commercially used probiotic functional products

Probiotic product	Manufacturer	Description
Activia	Danone, France	Creamy yogurt containing *Bifidus Actiregularis*
Actimel	Danone, France	Probiotic drinking yogurt with *L. casei* Immunitas
Aciforce	Biohorma, The Netherlands	Freeze-dried product containing *Bifidobacterium bifidum*, *L. lactis*, *L. acidophilus*, *Enterococcus faecium*
Bactisubtil	Synthelabo, Belgium	Freeze-dried product containing *Bacillus* sp. strain IP5832
Bactilac	THT, Belgium	Freeze-dried product with *Lactobacillus acidophilus*, *Lactobacillus rhamnosus*
Bififlor	Eko-Bio, The Netherlands	Freeze-dried product with *Lactobacillus acidophilus*, *Lactobacillus rhamnosus*, *Bifidobacterium bifidum*
Gefilus	Finland	A wide range of LGG products Valio
Hellus	Tallinna Piimatoostuse AS, Estonia	Dairy product containing *Lactobacillus fermentum* ME-3
Jovita Probiotisch	H&J Bruggen, Germany	Blend of cereals, fruit, and probiotic yogurt with *Lactobacillus* strains
Provie	Skanemejerier, Sweden	Fruit drink containing *Lactobacillus plantarum*
ProViva	Skanemejerier, Sweden	Refreshing natural fruit drink and yogurt in many different flavors containing *Lactobacillus plantarum*
Pohadka	Valasské Mezirıcí Dairy, Czech Republic	Yogurt milk with probiotic cultures
Proflora	Chefaro, Belgium	Freeze-dried product containing *Lactobacillus acidophilus*, *Lactobacillus delbrueckii* subsp. bulgaricus, *Streptococcus thermophilus*, *Bifidobacterium*
Revital Active	Olma, Czech Republic	Yogurt and drink yogurt with probiotics
Rela	Ingman Foods, Finland	Yogurts, cultured milk, and juices with *L. reuteri*
SOYosa	Bioferme, Finland	Range of products based on soy and oats and includes a refreshing drink and a probiotic yogurt-like soy–oat product
Snack Fibra	Celigueta, Spain	Snacks and bars with natural fibers and extra minerals and vitamins
Soytreat	Lifeway, USA	Kefir type product with six probiotics
Vifit	Campina, the Netherlands	Drink yogurts with LGG, vitamins and minerals
Vitality	Muller, Germany	Yogurt with pre- and probiotics and omega-3
Vitamel	Campina, the Netherlands	Dairy products with *Lactobacillus casei* GG, *Bifidobacterium bifidum*, *Lactobacillus acidophilus*
Yosa	Bioferme, Finland	Yogurt-like oat product flavored with natural fruits and berries containing *Lactobacillus acidophilus*, *Bifidobacterium lactis*
Yakult	Yakult, Japan	Milk drink containing *Lactobacillus casei* Shirota

### Health benefits of probiotics

**Fig. 1:**
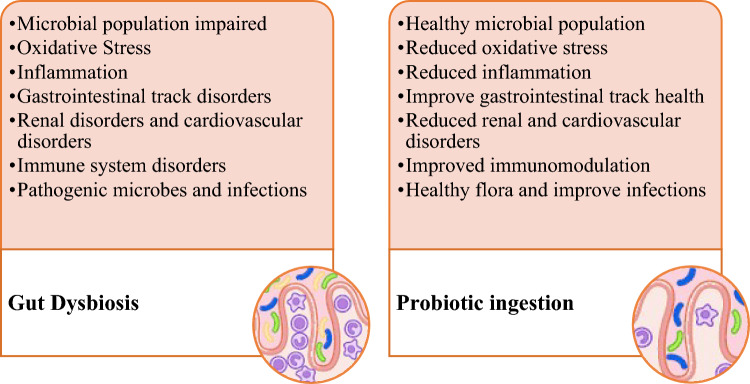
Altered gut microbial diversity and major health benefits produced by probiotic ingestion

Major health benefits produced by probiotic microorganisms are the establishment of favorable conditions of the intestine, a strong immune system, and a digestive system. Figure 1 demonstrates the different health benefits of probiotics. Probiotics have been shown to avert several inflammatory and allergic disorders (like rhinitis and atopic dermatitis), reduce the incidence of diarrhea, serve as antibiotics, control infections, and protect against bladder and colon cancers (De Prisco and Mauriello, 2016; Plaza-Diaz et al., 2019). Probiotics have been linked to prevent and/ or treat acute diarrheal infections, necrotizing enterocolitis, antibiotic-related diarrhea, pediatric colic, allergies, lactose intolerance, ulcerative colitis, *Helicobacter pylori* infection, Crohn’s disease, metabolic illnesses, neurological diseases, and respiratory tract infections (Liu et al., 2018; Télessy, 2019). Numerous clinical trials using probiotics have been successful to treat or alleviate symptoms of several diseases, for example, irritable bowel infection (Shadnoush et al., 2015), irritable bowel syndrome (Yoon et al., 2015), obesity (Song et al., 2020), infantile colic (Chau et al., 2015), Parkinson’s (Barichella et al., 2016), diabetes (Soleimani et al., 2017), rheumatoid arthritis (Zamani et al., 2016). Furthermore, in a study, *Lactobacillus rhamnoses* were encapsulated in a hydrogel that contains thiolated hyaluronic acid to treat Salmonella-induced enteritis. It was found that the encapsulated probiotic was more effective than free cells, suggesting that it could be a viable alternative to antibiotics (Xiao et al., 2020). Probiotics are widely in use among functional food products due to their proven health benefits and alternate treatment options for many diseases, some of which will be discussed in detail in this review.

### Impact on the age group

The fecal flora of children is different than in adults, disease such as gastroenteritis can alter the bacterial profiles in children. The frequency of dysbacteriosis is particularly high in children after antibiotic therapy. Probiotic functional foods can help to restore the microbial equilibrium of the intestine and control the side effects of antibiotic therapy. Adults, particularly older people frequently develop a condition called, atrophic gastritis, also called the incapability to secrete stomach acid. In the United States, almost one-third of older people are being affected by this condition. In atrophic gastritis, a lack of gastric acid can lead to the overgrowth of bacteria in the small intestine, which can influence micronutrient absorption (Zubillaga et al., 2001). The functional foods containing probiotics have the potential to regulate intestinal bacterial balance and enhance health outcomes within various demographic groups.

### Gastrointestinal (GIT) disorders

#### Allergy to food

Several research findings suggested that probiotic bacteria (*Lactobacillus rhamnosus* GG) may increase the mechanisms of endogenous barriers in individuals having food allergies and atrophic dermatitis. By reducing colonic inflammation, probiotics may be effective in the treatment of food allergies (Majamaa and Isolauri, 1997). When administering novel probiotic functional foods for treating GIT problems, the antigenicity level of the diet should be considered. Probiotics like *Lactobacillus rhamnosus* GG (LGG) can not only correct abnormal transport of macromolecules, but can also have a positive result on mucosal breakdown depending on the dietary antigen (Pessi et al., 1998). Functional food made of LGG is already available on the market and has proven health benefits, but it is imperative to conduct further evaluations to ensure the safety of these probiotics.

#### Diarrheal illness

It has been shown that pathogenic microorganisms such as *S. typhosa*, *S. dysenteriae*, and *E. coli* are inhibited by a fermented probiotic product comprising *L. acidophilus*. Probiotic administration in bacterial diarrhea produces a favorable impact by the production of antimicrobial compounds by *L. acidophillus*, that neutralize the enterotoxins produced by *E. coli* (Rani and Khetarpaul, 1998). *L. acidophilus* and *bifidobacteria* were found to reduce the development of colonic flatus in humans and the modulation of diarrhea caused by *Clostridium difficile* (Gallaher et al., 1996). Nine randomized and placebo-controlled trials have been conducted around the world for the prevention of diarrhea with different probiotic species, including, *Bifidobacterium lactis*, *Lactobacillus acidophilus Lactobacillus GG*, *Streptococcus thermophilus*, *Lactobacillus reuteri*, and *Lactobacillus rhamnosus*. They have demonstrated dose dependent efficacy to treat diarrhea (Guandalini, 2011). In another study, idiopathic diarrhea was improved by using probiotic *Escherichia coli* Nissle 1917 (EcN) (Rudinsky et al., 2023).

#### Lactose malabsorption

*Lactobacillus* supplementation is thought to improve lactose fermentation and alleviate lactose intolerance symptoms. According to studies, consumption of lactobacilli-containing food products can lower the activity of fecal bacterial enzymes such as nitroreductase, beta-glucuronidase, and azoreductase. Lactose malabsorption is adapted fast and metabolized in the small intestine by colonic flora. The study revealed that *L. acidophilus* improves in-vitro lactose fermentation when adaptation to lactose load by colonic flora is not developed and a stable bacterial population is not maintained. Lactose digestion is improved by *Lactobacillus acidophilus* strain LA-1, suggesting that the metabolic changes caused by supplements having *lactobacilli* can occur when a stable microflora has not been formed (Zubillaga et al., 2001). There are functional foods made of *Lactobacillus acidophilus* currently in human use.

#### *Helicobacter pylori* (*H. pylori*) infection

*Helicobacter pylori* is spiral-shaped and gram negative pathogen responsible for causing peptic ulcer and gastric malignancies (Patel et al., 2014). The association of diet with the occurrence of peptic ulcers has been linked. It has been demonstrated that people with confirmed peptic ulcers, showed a lower consumption level of fermented milk products and vegetables and a higher consumption level of bread, meat, and milk than ordinary individuals (Elmståhl et al., 1998). The lack of *Lactobacillus* specie in the stomach was discovered in patients with *H. pylori* infection. A decline in the *Bifidobacteria* population and an increase in opportunistic enterobacteria, as well as changes in local immunity, were found in *H. pylori-*infected population. The results suggested that using probiotic functional food which includes *lactobacilli* and *bifidobacteria* generated good outcomes for the treatment of immunological and microecological abnormalities. In children, *H. pylori* associated with gastroduodenal pathology can be treated with probiotics containing bifidobacteria and lactobacillus along with triple antibacterial therapy. The probiotic formulation is prescribed in the early phase of etiotropic therapy (Lykova et al., 1999). Based on many clinical studies, administration of probiotics combined with standard antibiotic therapy effectively treats *H. pylori* infection. Research reported that the use of *Lactobacillus casei*, *Lactobacillus rhamnosus* GG, *Lactobacillus reuteri*, and *Saccharomyces boulardii* can efficiently eliminate *H. pylori* infection (Keikha and Karbalaei, 2021). Probiotics are extensively used in therapeutic purpose for *H. pylori* infection as demonstrated in Table 3. However, the focus of future studies should be on probiotic species, optimal dosage, formulation in functional food, and length of treatment.

**Table 3:** Recent clinical advantage on animals and meta-analysis on the effect of probiotics in the treatment of *H. pylori* infection

Probiotic strain/strains	Animal/human subject	References
*L. rhamnosus* JB3	C57BL/6 mice	Chen et al. (2018)
*L. fermentum* UCO-979C	Mongolian gerbil	Merino et al. (2018)
*L. plantarum* ATCC8014	C57BL/6 mice	Afsahi et al. (2018)
*S. boulardii*	3592 patients	Zhou et al. (2019)
*Lactobacillus*	724 patients	Yu et al. (2019)
*Lactobacillus*	8924 patients	Shi et al. (2019)
*Lactobacillus*, *Bifidobacterium Saccharomyces*	525 patients	Pourmasoumi et al. (2019)
*L. fermentum* P2 (P2), *L. casei* L21 (L21), *L. rhamnosus* JB3 (JB3)	C57BL/6 mice	Lin et al. (2020)

### Immunomodulation

Probiotics have immunomodulatory properties which are strain specific. A study demonstrated that the phagocytic capability of the leucocytes isolated from blood samples of individuals who had previously consumed probiotics was boosted by *L. acidophilus* La1, which was consistent with the adhesion potential of the bacterium. However, even *Bifidobacterium lactis* Bb12, which has a somewhat lower adhesion, has been demonstrated to significantly improve phagocytosis. Furthermore, probiotics can stimulate IgA production by B cells. A noteworthy rise of IgA concentration in serum was observed in the subjects of the study with the intake of fermented milk containing *L. acidophilus* La1 and *Bifidobacterium bifidum*, following *Salmonella typhi* Ty21 vaccination. Additionally, children who received rotavirus vaccination and consumed *L. rhamnosus* GG alongside presented an increased quantity of IgA-secreting cells (Delcenserie et al., 2008). Probiotics are classified as either ‘immunostimulatory’ or ‘immunoregulatory,’ based on their capability to produce IL-12, hence augmenting the defense system of a host via the enhanced activity of NK cell and TH1 pathways, or ‘immunoregulatory,’ based on their capability to stimulate IL-10 and pathway of T-cell regulation. *Lactobacilli* often fall into the category of immunostimulatory, while *Bifidobacterium* generally falls into the category of immunoregulatory (Yaqoob, 2014). In another study, *Bifidobacterium breve* was used in the treatment of severe combined immunodeficient (SCID) mice, T cell-derived IL-10 inhibited T cell-dependent intestinal inflammation in mice (Jeon et al., 2012). In future their effect must be evaluated in well-designed clinical research.

### Anti-cancer activity

Cancer is the deadly disease worldwide. The administration of probiotic supplementation has been proven in animal trials to prevent the formation, development, and metastasis of chemically induced and transplantable tumors. Probiotic therapy has also been shown to decrease the occurrence of colonic cancer by preventing the transformation of procarcinogens to active carcinogens, production of antimutagenic compounds, inactivation of mutagenic compounds, suppression of pro-carcinogenic bacterial growth, immune function enhancement, reducing mutagens absorption of from the intestine, anti-proliferation by cell differentiation and the regulation of apoptosis, undigested food fermentation which aids in generating short-chain fatty acids (SCFA), and inhibiting signaling pathways for tyrosine kinase (Gill and Guarner, 2004; Uccello et al., 2012). The results of many clinical studies indicate the effectiveness of probiotics in prevention and treatment of many cancers including breast, cervical, colon, liver, gastric, pancreatic, bladder, and colorectal cancer (Table 4).

**Table 4:** Examples of probiotics in treatment of different types of cancer

Cancer type	Probiotic strain/strains	References
Breast cancer	*Lactobacillus brevis* MK05	Pourbaferani et al. (2021)
Colorectal carcinoma	*Lactobacillus plantarum* CGMMCC No 1258, *Lactobacillus acidophilus* LA-11, *Bifidobacterium longum* BL-88	Liu et al. (2011)
	*Bifidobacterium longum*, *Lactobacillus acidophilus*, *Enterococcus faecalis*	Zhong et al. (2014)
	*Bifidobacterium lactis* Bb12, *Lactobacillus rhamnosus* GG	Rafter et al. (2007)
	*Bacillus natto*, *Lactobacillus acidophilus*	Ohigashi et al. (2011)
	*Lactobacillus casei* BL23	Jacouton et al. (2017)
	*Lactococcus lactis* subsp. lactis isolate (R7)	Jaskulski et al. (2020)
Cervical cancer	*Lactobacillus acidophilus*, *Bifidobacterium bifidum*	Chitapanarux et al. (2010)
Colon carcinogenesis	*Lactobacillus casei*	Irecta-Nájera et al. (2017)
	*Lactobacillus plantarum* YYC-3	Yue et al. (2020)
	*L. rhamnosus strain* Y5	Dehghani et al. (2021)
Gastric cancer	*Lactobacillus kefiri* P-IF	Ghoneum and Felo (2015)
	*Lactobacillus reuteri* PTCC 1655	Rasouli et al. (2017)
	*Lactobacillus plantarum* MH-301, *L. rhamnosus* LGG-18, *L. acidophilus* and *Bifidobacterium animalis* subsp. lactis LPL-RH	Zheng et al. (2021)
Liver cancer	*Lactobacillus rhamnosus* LC705, *Propionibacterium freudenreichii* subsp. shermanii	El-Nezami et al. (2006)
Lung cancer	*Lactobacillus acidophilus*	Gui et al. (2015)
Pancreatic cancer	*Lactobacillus paracasei* GMNL-133 *and Lactobacillus reuteri* GMNL-89	Chen et al. (2020)

## Hypercholesterolemia

Hypercholesterolemia is defined as metabolic syndrome associated with abnormal serum and cellular cholesterol levels (Kumar et al., 2012). Cholesterol is significant biological compound, but the high serum cholesterol levels have a direct role in many health disorders, such as, hypertension, coronary heart disease, diabetes type II, atherosclerosis (Chien et al., 2010). Existing evidence suggests that the management of cholesterol decreases the chance of cardiovascular disease or reduces its progression. Previously, reducing the level of low-density lipoprotein (LDL) cholesterol in plasma was restricted to changes in diet and the use of medications such as statins. Recently, probiotic functional food are in the growing demand for the control of cholesterolemia (Poli et al., 2018). Daily administration of *L. rhamnosus* GG in a concentration of 1 × 10^8^ CFU per mouse, for 13 weeks in dyslipidemic mice helped to restore the microbiota of the gut, and showed improvement in hypercholesterolemia, hepatic fat accumulation, and hypertriglyceridemia (Kim et al., 2016). In another study, *L. rhamnosus* hsryfm1301 induced for 28 days in 10^9^ CFU/mL, reduced the level of cholesterol and triglycerides in the serum of a hyperlipidemic rat model (Chen et al., 2014). Similarly, consumption of *L. acidophilus* NS1 for 10 weeks in a concentration of 1.0 × 10^8^ CFU/mL, on a long-term daily basis reduced LDL-cholesterol, plasma cholesterol, and triglycerides levels in a diet-induced obese model of mice (Song et al., 2015). Moreover, in diet-induced hypercholesterolaemic rats, a daily intake of a mixture of probiotics containing *B. lactis*, *B. longum*, *B. brevis*, *L. plantarum*, and *L. reuteri* in different doses for 8 weeks, lowers the LDL-cholesterol, total serum cholesterol, triglycerides, and prevent hepatic steatosis (Kim et al., 2017). Clinical findings on a controlled, randomized, double-blind trial suggest that *L. plantarum* CECT 7527, CECT 7528, and CECT 7529 supplementation for 12 weeks decreases the level of cholesterol in hypercholesterolaemic patients (Fuentes et al., 2013). Furthermore, *L. acidophilus* L1 and probiotic yogurt made by fermentation with a starter culture of *B. lactis* and *L. acidophilus*, when supplemented with hypercholesterolemic subjects showed a reduction in cholesterol level (Anderson and Gilliland, 1999; Ataie-Jafari et al., 2009). Recently, a meta-analysis with clinical trials confirmed that consumption of fermented milk products supplemented with probiotics and supplements containing probiotic bacteria effectively reduces LDL-cholesterol and total cholesterol levels in serum (Shimizu et al., 2015). Likewise, another study proved that ingestion of probiotics affects reducing LDL-cholesterol and total cholesterol in human subjects with high cholesterol, borderline high levels, and a normal level of cholesterol (Guo et al., 2011). Probiotic functional foods play a significant role in regulating lipid metabolism and have a therapeutic effect on dyslipidemic disorders. Further large-scale studies on clinical subjects must be conducted.

### Diabetes mellitus type 2 (DMT2)

Diabetes mellitus Type 2 is a common metabolic illness, and it occurs when the pancreatic cells are unable to produce sufficient insulin for maintaining a normal level of glucose in the blood, or when the pancreatic cells become resistant to insulin (Boada and Martinez-Moreno, 2013). Associated risk factors are age, physical inactivity, diet, obesity, genetic susceptibility, and sedentary lifestyle (de Almeida-Pititto et al., 2015; Hu et al., 2001). Current evidence proposes that disturbance of intestinal permeability and dysregulation of gut microbiota could also be linked to DMT2 development (Cani et al., 2012; Flint et al., 2012; Gerritsen et al., 2011; Navab-Moghadam et al., 2017). Disruption in gut microbiota cause changes that disrupt pancreatic cells, reduce insulin resistance, and develop DMT2 (Ly et al., 2017). Studies suggest that probiotic formulations can restore gut permeability and microbial balance. They can also reduce pro-inflammatory markers, reactive oxygen species, and insulin resistance (Wang et al., 2017a, 2017b). An experimental finding indicates that long-term intake of 0.1 g probiotic lyophilized powder having *Bacillus subtilis*, *L. reuteri*, and *L. crispatus* for 8 weeks in the concentration of 10^10^ CFU/mL/day, reduced the HbA1c and plasma glucose levels. It also improved the oral glucose tolerance test and in adipose tissues, Glut-4 mRNA is up-regulated in the streptozotocin-induced diabetic rats (Memarrast et al., 2017). Moreover, consuming a mixture of probiotics having *L. plantarum*, *L. rhamnosus*, *L. casei*, *L. plantarum* CCFM36, and *L. breve* for 10 weeks with a dose of 1.6 × 10^10^ CFU/day, efficiently decreased leptin levels and HbA1C, enhanced insulin resistance, glucose tolerance and protected against the pancreatic impairment in diabetes type 2 mice (Li et al., 2016). Human studies in placebo-controlled, small-scale, double-blind groups have also revealed the therapeutic impact of the probiotic product on DMT2. Another meta-analysis in a double-blind, randomized placebo-controlled trial for 12 weeks was carried out, probiotics were supplemented in a dose of 3 × 10^10^ CFU. Six viable cell probiotics including *L. casei*, *L. acidophilus*, *L. lactis*, *B. longum*, *B. bifidum*, and *B. infantis* were administered to 136 patients with no insulin-dependent diabetes separated into a probiotic group (37 males & 31 females) and a placebo group (34 males & 34 females). It improved fasting glucose levels and serum HbA1c in both genders Recent meta-analyses of randomized clinical studies supported the results and confirmed that the probiotic formulation is efficiently related to an improved HbA1c level and fasting insulin in DMT2 patients(Akbari and Hendijani, 2016). Another 8 weeks of randomized controlled clinical findings demonstrated improved factors related to oxidative stress, for example, glutathione reductase and glutathione peroxidase levels in 48 patients with diabetic kidney disease. They were administered a 200 mL *L. plantarum A7* per day probiotic with soy milk (Miraghajani et al., 2017). Furthermore, in a different clinical study, multi-probiotic species were administrated containing freeze-dried and viable strains of *L. bulgaricus*, *L. casei*, *L. acidophilus*, *L. rhamnosus*, *B. longum*, *B. breve*, and *Streptococcus thermophilus* in different concentrations for 8 weeks, in association with 100 mg fructo-oligosaccharide in Iranian diabetic patients. It showed improvement in the total antioxidant capacity and total glutathione levels (Asemi et al., 2013). The outcome of these findings makes probiotics a perfect therapeutic candidate to be involved in functional food.

### Chronic kidney disease

Renal impairments can cause cardiovascular disorders through several mechanisms. Several factors that cause renal abnormality are traditional factors such as hypertension, diabetes, and dyslipidemia. The non-traditional factors include hemodynamic and metabolic abnormalities caused by a renal abnormality (e.g., oxidative stress and inflammation) that can impact the development of cardiovascular disorders in patients with chronic kidney disease (Longenecker et al., 2002). Research has found that the composition of microbiota in patients with chronic kidney disease is altered, and the communication between gut microbiota and the host is a key event in the pathophysiology of the disease. Whereas uremia impacts both the composition and metabolism of gut microbiota, significant uremic toxic compounds are produced by the metabolism of gut microbiota, suggesting that this process is bi-directional. These toxins are eliminated by the kidneys, primarily through tubular secretion, and are hence classified as uremic toxins (Poesen et al., 2013). Intestinal bacteria digest about 10 g of protein every day in the colon, converting it to metabolites like ammonium, amines, phenols, indoles, and thiols. Most of these products are removed through feces; however, the kidneys are responsible for a portion of their absorption and disposal. The accumulation of these products is caused by impaired renal function, as seen in CKD (Evenepoel et al., 2017; Khoury et al., 2017). Moreover, disruption of the gut microbiota damages the epithelial barrier integrity, which has been linked to gut ischemia and intestinal wall edema, the result is increased endotoxin exposure of the host tissues, together with the kidneys (Vaziri, 2012). Emerging approaches to restoring the intestinal environment during the progression of CKD include the administration of probiotics (Koppe et al., 2015). Genetically modified microencapsulated live cells from the urease-producing bacteria *E. coli* DH5 were orally administered to uremic rats and significantly decreased the plasma urea levels (Prakash and Chang, 1996). In a different study, a casein-based food diet with added probiotics Sporlac (*L. sporogenes*, 1 × 10^8^ CFU per day), *Bacillus pasteurii* (1 × 10^9^ CFU/day), kibow cocktail (containing *Bifidobacterium* spp*.*, *L. acidophilus*, *L. bulgaricus*, *L. reuteri*, *L. casei*, *S. thermophilus* at 1 × 10^10^ CFU/day) or *S. boulardii* (1 × 10^9^ CFU/day) was fed to a chronic renal failure model (nephrectomized rats) for 16-weeks trial and reduced blood urea nitrogen (BUN) levels and extended life were observed (Ranganathan et al., 2005). Clinical studies found that the intake of *L. acidophilus* orally decreased the levels of intestinal toxins such as nitroso-dimethylamine, and dimethylamine (DMA), which are produced in the plasma of patients on hemodialysis (Simenhoff et al., 1996). The results were supported by another study in which, a double-blind pilot study was conducted on 46 diseased individuals in stage III or IV of chronic kidney disease. A mixture of probiotics named KB (*B. longum KB31*, *L. acidophilus KB27*, and *S.thermophilus KB19*, in a dose of 10^9^ CFU per day) was given for a duration of six months. A reduction in BUN was observed together with improved quality of life (Ranganathan et al., 2010). A clinical trial on stages 3 and 4 of disease patients was carried out and a commercially available lyophilized symbiotic named Probinul-neutro®, containing *L. casei* subsp. Rhamnosus, *L. plantarum*, *L. gasseri*, *L. acidophilus*, *L. sporogenes*, *L. salivarius*, *B. longum*, *B. infantis*, and *Streptococcus thermophilus* was given, along with prebiotic inulin tapioca-resistant starch was suggested for 4 weeks in the dosage of 5 g × 3/day. This supplementation reduced the level of plasma p-cresol (Guida et al., 2014). This indicates the therapeutic potential of probiotics in chronic kidney disease patients but there is no defined dosage, intervention timeframe, mechanism, and pathways to define probiotic efficacy in this regard. So, further research is needed to explore the full potential of probiotics in reducing chronic kidney disease (Neto et al., 2018).

### Heavy metal intoxication

The typical treatment method against toxicity produced by heavy metals was dependent on chelation therapy, which uses different chemical chelators. Chelators have several side effects, including cardiac arrest, kidney overload, anemia, and mineral insufficiency (Flora and Pachauri, 2010). Recently, probiotics, nanoparticles, essential amino acids, folate, and vitamins C and E have all been used as treatment candidates for heavy metal intoxications (Duan et al., 2020; Inbaraj and Chen, 2012; Yang et al., 2019).

New functional food was proposed in a study composed of selenium nanoparticles (SeNPs) generated by the means of green synthesis utilizing *L. casei.* For the first time, its capacity was investigated for liver injury bioremediation. The ability of SeNPs to defeat cadmium-induced hepatic toxicity was also checked. Elemental SeNPs exist in two forms; purified SeNPs and lactic acid bacteria (*L. casei*) together with endogenous SeNPs (called LSeNPs). They were investigated in mice with liver toxicity induced by cadmium. LSeNPs were given orally for 30 days in a dosage of 0.4 mg/kg b.w. Treatment with both SeNPs forms ameliorated Cd-induced liver damage in the mouse. Results presented a decrease in pro-apoptotic *bax*, including a rise in the expression of anti-apoptotic *bcl-2*. Also, a reduction in the gene expression of inflammatory markers of the liver was observed. LseNPs showed excellent hepatoprotective effects. A functional diet containing both elemental SeNPs and probiotic bacteria might be used to eliminate liver damage caused by cadmium and improve the nutritional potential and health benefits. A potential new technology for the food industry is to create yogurt enhanced with LSeNPs with heavy metal remediation properties (Vicas et al., 2021). Further clinical studies must be determined to support the effectiveness of this in functional food products.

### Microencapsulation of probiotics

Most of the functional foods formulated with probiotics are available in the dairy industry. Many available foods in the market that are incorporated probiotics are supplemented with the free microbe, and just a few use their microencapsulated forms (De Prisco and Mauriello, 2016). Besides their function in boosting the health of individuals upon intake, probiotics could also be integrated into edible polymeric matrices to produce bioactive packaging of food. They can substitute antibiotics by suppressing spoilage and infective bacterial species, also improving the safety of food (Espitia et al., 2016). The key functional food products having probiotic microcapsules are bakery products like cakes, bread, and biscuits; Dairy products like cheese and yogurt; vegetables and fruit juices; meat products like fermented sausages; and others include mayonnaise, ice cream, and fermented beverages. Table 5 shows several examples of microencapsulated probiotic bacterial species and the method used for encapsulation in food products (Burgain et al., 2011; De Prisco and Mauriello, 2016; Terpou et al., 2019). Probiotic bacteria can be co-encapsulated with biologically active materials such as prebiotics, antioxidants, curcuminoids, and omega-3 fatty acids within the same matrix and subsequently integrated into food, making it an afunctional product. The bioactivity of both is increased and it can also show synergistic health benefits to the host. Appropriate wall materials are required to achieve such synergistic effects, and their functioning must be improved (Misra et al., 2021). Functional products of these types are generally commercialized in powdered form, chewable tablets, or capsules. The process of encapsulation could be achieved through a technique called “spray coating”. In this technique, fat-based polymers are sprayed on a probiotic microorganism to coat them, which increases their availability in the environment of the gastrointestinal system. Commercialized products are Probiocap™ and STAR™ technologies (Kwak, 2014). *L. casei* was added to juices of citrus fruits in the free form and microencapsulated form by using the vibration technique. Their viability was evaluated at 4 °C after 28 days based on the fruit juice type, the low pH had an impact on bacterial survival during storage. A few microcapsules started to break in the pineapple juice, but they showed recovery after 28 days with 100% viability (2.3 × 10^7^ CFU/g spheres). The orange juice showed greater than 90% viability (5.5 × 10^6^ CFU/g spheres). Though, raspberry juice showed a rapid drop in viability, eventually vanishing at the end of storage, possibly due to the absorption of anthocyanin inside microcapsules. Another study demonstrated the protection of thermotolerant lactic acid bacteria against pasteurization in cooked sausages, ionotropic alginate-pectin gels were used to co-encapsulate them with the prebiotics produced by residues from agriculture and industry (Barragán‐Martínez et al., 2020). Sausages had good sensory qualities, a higher count of lactic acid bacteria, the pathogenic bacteria were inhibited, and the synergetic prebiotics use reduced oxidative rancidity of fat (Reque and Brandelli, 2021).

**Table 5:** Microencapsulation of probiotic bacteria and their application in food products

Probiotic	Product	Encapsulating material	Encapsulation method
*B. longum*	Cheddar cheese	Na-alginate and palmitoylated alginate	Droplet extrusion and emulsion method
*B. animals*	Kefir	Sodium alginate	Extrusion
*B. bifidum*, *B. infantis*	Mayonnaise	Alginate	Emulsification
*B. longum*	Yogurt	k-Carrageenan	Emulsification
*L. acidophilus*	Carrot juice	Alginate-inulin xanthan gum	Extrusion
*L. rhamnosus*	Apple juice	Whey protein isolate and in combination with modified resistant starch	Spray drying
*L. helveticus*, *B. longum*	Chocolate	Fatty acids	Spray coating
*L. acidophilus*, *B. infantis*	Cheddar cheese	Alginate/starch	Emulsification
*L. casei* ATCC393	Fermented milk	Chios mastic gum	Freeze drying
*L. reuteri*	Dry fermented sausages	Alginate	Extrusion
*L. plantarum* and *B. longum*	Fruit juice	Alginate or pectin coated with chitosan, glucomannan or gelatin	Extrusion
*L. rhamnosus*	Fruit juice	Whey/alginate	Droplet extrusion with coating via electrostatic deposition
*L. acidophilus*	Tomato juice	Ca-alginate	Extrusion
*L. casei* LC-01 and *B. lactis* BB-12	Ice cream	Alginate and maize resistant starch	Emulsion
*L. acidophilus* LA-5	Yogurt	Pectin/whey protein	Ionic gelation and complexation
*L. plantarum*	Mango juice	Calcium alginate/soy protein isolate	Gelation
*L. acidophilus*, *B. bifidum*	White brined cheese	Alginate	Extrusion and emulsification
*L. paracasei* ssp. paracasei LBC-1	Mozzarella cheese	Alginate	Extrusion
*L. acidophilus*	Yogurt	Alginate and chitosan	Extrusion
*L. acidophilus*, *B. longum*	Yogurt	Maltodextrin/gum Arabic	Spray drying

Data compiled from (Burgain et al., 2011; De Prisco and Mauriello, 2016; Terpou et al., 2019)

In the most recent studies, the spray drying technique was used to prepare a powdered functional food made up of carbohydrate polymers and active ingredients. *Bacillus clausii* as a probiotic, and Quercetin as an antioxidant were co-microencapsulated using carrying agents; inulin (IN) and maltodextrin (MX). The IN-MX blends had a synergistic effect on viability and antioxidant activity. The microbial viability improved by adding MX, whereas antioxidant activity was improved by adding IN. A surface response plot revealed that the yield was highly dependent on temperature drying and then the concentration of IN. This study demonstrated the benefits of using combinations of carbohydrate polymers for the preservation and microencapsulation of active compounds for applications in functional foods and pharmaceutical use (Saavedra-Leos et al., 2022). The use of probiotics in functional food via the microencapsulation technique has a plethora of benefits because of its advantage in protecting a functional component. It can help in reducing the global shortage of micronutrients and has a promising future (Palanivelu et al., 2022).

## Discussion

Functional foods are by far the most rapidly growing division of the food and beverage industry with a promising future. Intestinal microbiota research is gaining attention in a variety of fields. Various research studies give evidence for the use of probiotics in food for obtaining health advantages. The significance of using gut microorganisms in the production of functional foods is a major area of research. The evidence from scientific data suggested that disturbance of the gut microbial environment leads to multiple diseases, and intake of functional food based on probiotics demonstrated beneficial health effects in so many ways. However, there are still some knowledge gaps in most of the studies as there is limited information on the safety of probiotics in many pieces of research. More evidence is required for the survival of probiotics in functional food for the duration of its shelf life. Furthermore, food scientist needs to focus on the selection of the most appropriate strain for the combination of probiotics in the food matrix, which is an essential component. Also, convenient packaging and storage conditions should be provided to produce effects in the host, this area should be explored deeply. After a deep evaluation of studies conducted on the properties of probiotics, the concluding results are still controversial, and less research has been carried out on human subjects. Further research on the assessment of probiotics in the prevention of diseases is a need and more large-scale clinical research must be performed. Finally, the area of consumer awareness must be focused on to achieve maximum success in the probiotic functional food market.

## Future direction

Functional food is currently involved in extensive scientific research to fully explore this area of study. Globally, government sectors, and academic, and private research institutions are dedicating significant efforts to identify how food ingredients and functional foods might help prevent disease and/or infection, improve health, lower the cost of healthcare, and enhance the standard of health for people. Nutrigenomics is a developing field in which the link between diet and disease development is analyzed depending on a genetic profile of an individual. In this, a personalized functional diet can be prescribed using an individual’s exact genetic profile. It is going to have a significant impact on the future of new probiotic functional food products for the prevention of disease (Hasler, 2002). The next-generation probiotics shall be based on population-level strategy and/ or personalized nutrition strategies in which novel probiotic formulations could be made, consisting of indigenous gut bacteria that will be effective on humans.

## Data Availability

Not applicable.
